# A CURE for a Major Challenge in Phenomics: A Practical Guide to Implementing a Quantitative Specimen-Based Undergraduate Research Experience

**DOI:** 10.1093/iob/obaa004

**Published:** 2020-02-20

**Authors:** S A Price, O Larouche, S T Friedman, K A Corn, P C Wainwright, C M Martinez

**Affiliations:** 1 Department of Biological Sciences, Clemson University, Clemson, SC 29634, USA; 2 Department of Evolution and Ecology, University of California—Davis, Davis, CA 95616, USA

## Abstract

The measurement and analysis of phenotypes is often a rate-limiting step for many integrative organismal studies but engaging undergraduate researchers can help overcome this challenge. We present a practical guide to implementing a quantitative specimen-based Course-based Undergraduate Research Experience (CURE), which trains students to collect phenotypic data and mentors them through the entire scientific process using the data they help to collect. Direct access to specimens is not necessary to implement this undergraduate research experience, as recent efforts to digitize museum collections along with online image archives allow data extraction to take place in any classroom. We focus in particular on hypothesis development and quantitative skills, as they are essential for modern biological discovery but are rarely emphasized in traditional lecture-based classes. We have implemented this experience, focusing on collecting and analyzing body shape data across fishes, at two institutions with a total of 39 students. It has so far resulted in 14 talks and 4 posters presented by students at local symposia and 2 scientific papers in preparation with undergraduate co-authors. Moreover, the students had a positive experience that, according to their own assessment, improved their critical thinking and analytical skills as well as their knowledge of science and the scientific process.

## French synopsis

La caractérisation et l’analyse de phénotypes peuvent imposer des contraintes temporelles importantes dans le cadre d’études intégratives sur la biologie des organismes. Une solution avantageuse pour pallier ce problème est de solliciter la participation d’étudiant(e)s-chercheur(e)s de premier cycle universitaire. Nous proposons un guide pratique afin de mettre en œuvre une expérience de recherche par cours pour des étudiant(e)s de premier cycle (CURE = Course-based Undergraduate Research Experience) fondée sur l’analyse quantitative de spécimens. Au cours de cette expérience, nous entraînons les étudiant(e)s à récolter des données phénotypiques qui sont ensuite utilisées afin de leur enseigner l’ensemble des étapes du processus scientifique. Considérant les efforts récents investis dans la digitalisation de collections muséales et la disponibilité de bases de données d’images en ligne, l’accès direct à des spécimens n’est pas fondamental à la complétion de cette expérience de recherche, les données nécessaires pouvant être extraites d’internet dans n’importe quelle salle de classe. Nous focalisons particulièrement sur l’élaboration d’hypothèses et le développement d’aptitudes en analyses quantitatives, puisque ces compétences sont essentielles aux découvertes contemporaines en sciences biologiques malgré qu’elles ne reçoivent généralement que peu d’emphase dans les formations traditionnelles dans le domaine. Nous avons réalisé cette expérience dans deux institutions universitaires avec un total de 39 étudiant(e)s afin de récolter et d’analyser des données morphologiques à travers l’ensemble des poissons. Jusqu’à présent, les résultats de ces analyses ont fait l’objet de 14 présentations orales ainsi que 4 affiches scientifiques dans des conférences régionales, et nos étudiant(e)s de premier cycle seront co-auteur(e)s de 2 articles scientifiques en cours de préparation. De plus, les étudiant(e)s ont apprécié cette expérience et ont affirmé avoir vu une amélioration dans leur capacité à exercer une pensée critique, dans leurs aptitudes analytiques, ainsi que dans leurs connaissances de la science et du processus scientifique.

## Introduction

Phenomics, the measurement and analysis of a large set of phenotypes, is necessary for many integrative organismal studies. It is particularly important for evo-devo and genomic studies, as the phenotypes of hundreds to thousands of individuals need to be measured to link genotype to phenotype (Houle 2010; Houle et al. 2010). Similarly, phenotypic data on hundreds to thousands of species are required to address evolutionary and ecological questions across large temporal, spatial, and phylogenetic scales (Chang and Alfaro 2016). The rate-limiting step for all of these studies is the amount of time it takes to collect the phenotypic data (Muñoz and Price 2019) but engaging undergraduate researchers can help overcome this challenge. However, training students to reliably collect phenotypic data takes time and requires commitment by both the undergraduate and senior researchers. We therefore developed a Course-based Undergraduate Research Experience (CURE) that not only trained students to collect large amounts of data but also mentored those same students to develop, test, and present scientific hypotheses using the data. This means that students were an integral part of the scientific discovery as well as the data collection, ensuring their investment in the success of the project. Moreover, pedagogically we were able to provide students with the important but rare opportunity to learn the scientific process through practice (Seymour et al. 2004; Lopatto and Tobias 2010). In this paper we present a practical guide to implementing a quantitative specimen-based CURE. We emphasized hypothesis development, analytical skills, and the presentation of results, as they are rarely covered in traditional lecture-based classes but are essential aspects of modern biological science.

It is important to note that direct access to specimens is not necessary to implement this undergraduate research experience. This may be particularly useful for biologists at teaching institutions that may have limited research infrastructure and budgetary support. Recent efforts to digitize museum collections (e.g., Integrated Digitized Biocollections www.iDigBio.org), along with online data archives for 3D scans (e.g., www.morphosource.org) and other phenotypic data including 2D images (e.g., www.morphobank.org) enable data extraction to take place in any classroom. Data can be obtained from images using free software available online such as ImageJ (Rasband 1997–2018) for linear measurements or tpsDIG2 (Rohlf 2015) and the R packages geomorph (Adams et al. 2018) and stereomorph (Olsen and Westneat 2015) for geometric morphometric approaches. In addition, shorter classroom research experiences, emphasizing hypothesis development and testing, could be developed using existing phenotypic datasets. We provide some additional resources in Online Appendix S1.

It is well established that research experiences for undergraduates are critical for providing a better understanding of the scientific process, promoting the development of critical thinking skills and self-confidence as well as creating pathways to science careers (e.g., Seymour et al. 2004; Lopatto and Tobias 2010). These effects are magnified for women and other students from underrepresented groups (Gándara and Maxwell-Jolly 1999; Barlow and Villarejo 2004; Villarejo et al. 2008). Traditionally, to gain research experience undergraduates “intern” in a laboratory and receive individual mentorship from a graduate student, postdoctoral researcher, or faculty member but this severely limits the number of students exposed to research (Wood 2003; Desai et al. 2008; Harrison et al. 2011). Therefore, CUREs, which engage an entire class in a research question (Wei and Woodin 2011), have become increasingly popular (e.g., Oufiero 2019) due to the increased accessibility of research opportunities to a larger number of students (Rowland et al. 2012). According to Auchincloss et al. (2014) CUREs are defined by the use of scientific practices, the process of discovery, the potential to contribute relevant scientific knowledge to the field, collaboration among students and mentors, and the ability to revise and repeat analyses based on initial results. In other words, the students do actual science. CUREs are typically added to the regular undergraduate curriculum and implemented like any other class, meeting several times a week for a few hours. Our implementation used an extended format, allowing the students to take our CURE course in addition to their regular class schedule, as our participants were from several different majors and departments. We met for just 60–90 min a week over an 18-month period, giving a total classroom time of somewhere between 40 and 60 h, which is roughly equivalent to most single semester CUREs. We have run this experience at two institutions with a total of 39 students, divided into three separate cohorts starting at 12-month intervals.

We developed this CURE to recruit undergraduate researchers to aid in measuring specimens of thousands of fish species, enabling us to generate a morphospace of body shapes across teleostean fishes (Price et al. 2019). There are about 31,000 species of teleost and we aimed to sample broadly across the phylogeny measuring and photographing up to 20% of the valid, living species. This was an enormous undertaking, over the past 3 years (summers 2016, 2017, and 2018) we spent a total of 7 months at the Smithsonian National Museum of Natural History and during this time 27 researchers participated in the data collection, two-thirds of whom were undergraduates. Our back of the envelope calculations estimate that we invested over 6000 person-hours in the generation of the dataset at the museum, measuring and photographing a total of 16,609 specimens from 6144 species across 394 families. While machine learning holds promise for rapid and automatic phenotyping (Macleod 2017), recent studies that have generated large phenotypic datasets for macroevolutionary analyses have focused on crowdsourcing data collection from images or scans using the general public either in the form of volunteers (https://www.markmybird.org/ see Cooney et al. 2017) or people receiving micropayments through websites like Amazon Turk (Chang and Alfaro 2016). However, we believe that working with a team of undergraduate researchers benefited our scientific goals in several ways not possible with crowdsourcing or machine learning approaches. Firstly, it allowed us to take a large team of researchers to a museum where photographs and measurements could be taken directly from specimens. Direct measurements on specimens provided a dataset in three dimensions available for immediate analysis and photographs resulted in a 2D image dataset for future analysis. Previous studies either focused on scanning the specimens and then processing the images (Cooney et al. 2017) or were constrained by the taxonomic samples of pre-existing image data collections (Chang and Alfaro 2016). Secondly, we were able to carefully select and fully train our data collectors and supervise the data collection, encouraging them to ask for advice if they were having problems identifying anatomical structures. Thirdly, our data collectors were invested in the data quality, as they were using it to conduct their own scientific research. Finally, it increased our scientific productivity, as the students gave talks and posters at local symposia and some have also been involved as coauthors on two scientific papers that are currently in preparation for publication. While our research focused on data collection across many species to address macroevolutionary questions, the generation of large phenotypic datasets provides opportunities for discovery across a broad spectrum of ecological, evolutionary, and organismal research.

## Methods

### Recruiting a diverse research team

Every research team should aim to recruit a diversity of students to broaden participation, promote inclusion, and enhance team performance. We targeted students in their first or second year and used several strategies to recruit from a broad pool. Following recommendations for recruiting women and underrepresented groups (see review by Ahmad et al. 2019) adverts encouraged students of all backgrounds to apply to join our research team, the application process was short, requiring just a CV and an email saying why they were interested, and listed few requirements except enthusiasm for experiencing research. We also explicitly mentioned it was ideal for students with no previous research experience. Adverts highlighted the possibility of being invited to participate in paid summer research, emphasizing that the museum experience was not just available to those that could afford to volunteer for a month. We generated a broad audience for the advert by printing it out and placing it around life sciences buildings, sending it to groups on campus that work with under-represented and first generation students in STEM and asking instructors in the Introductory Biology series and/or Vertebrate Biology to send out the advert to all of their students.

The principal investigator (PI) and usually one graduate student mentor met in-person with every applicant for 20–30 min. We described the project, explaining both the science and the vision for the team to hopefully excite the student’s interest and set them at ease. This was followed by a discussion about their general and specific interests. Offers were made based on their meeting and were not based purely on their application, GPA, and test-scores, a practice which may also improve the recruitment of under-represented candidates (e.g., Gándara and Maxwell-Jolly 1999). The students were ranked by their enthusiasm, potential, and their availability for the duration of the program. As all people have implicit biases (e.g., Staats 2016) that can negatively influence the recruitment of diverse undergraduates, the rankings were explicitly reevaluated by asking ourselves what the potential sources of bias may be for each candidate and whether any of our comments or rankings may have been influenced by them. If we determined that our perceptions may have been biased, the rankings and comments were amended. This personal interrogation of our assessments did lead to several changes in ranking. We aimed to recruit approximately twice the number of students that we needed to participate in the data collection each summer, this allowed for students choosing not to participate due to other commitments or lack of interest, as well as providing us with the opportunity to identify and invite the students that were best suited to the work at the museum.

### Promoting student ownership

Student ownership is important for individuals persisting in science and medicine (Hanauer et al. 2012). To promote ownership students were recruited to become members of our research team (in contrast to just taking a class) and throughout we emphasized that they were integral to the success of our overall scientific mission. The students were told that the mentors (faculty, postdocs, and graduate students) were there to guide, train, and inform but not to direct them. The students understood that their primary goal was to work together to develop and test a scientific hypothesis suitable for publishing as a scientific paper, using the fish body shape data they were helping to collect. However, they were also made aware that there are a lot of variables outside of our control that may prevent the publication of their findings. Students were also involved on a more practical level with the organization of the research. The students identified when the team would meet, finding a time that worked with all of their schedules. Through discussion they also helped to set major deadlines throughout the experience, such as the date on which they would pick the hypothesis and when they wanted to have a draft of their presentations ready for feedback.

### Engaging in the process of science

The process of science involves asking questions, developing hypotheses and making predictions, collecting data with which to test the predictions, and then re-evaluating the hypotheses, predictions, data, and analyses in light of the findings. Every student recruited to the CURE participated in this process by progressing through four equal sections: (A) data collection training, (B) hypothesis development, (C) analytical methods, and (D) interpretation, evaluation, and presentation. The body shape data collection at the museum occurred between sections A and B, it was an extra “paid internship” that was neither guaranteed nor required for participation in the CURE. The timeline differed slightly for each institution, as one is on the quarter system and the other has semesters (see Online Appendix S2). Data collection training came first out of necessity, as we needed our team trained and ready to collect data in the summer. However, we still recommend this sequence, as it enabled the students to gain some background knowledge prior to embarking on hypothesis generation. Through the data collection training they became more familiar with the diversity of their study organisms, the data, and some of the generally expected relationships between ecology and morphology. Grading depended on the institution and the way we were able to implement a course that allowed students to receive both research and class credit (each semester was a total of two credits). We essentially treated it as a pass–fail class, if full grades were required a student received an A if they participated by attending class and submitting the required work.

It should be noted that not every student recruited finished the four sections, some attrition is inevitable when requiring such a long-time commitment. Each year we lost one student after completing the first semester/quarter usually due to scheduling conflicts or the need to focus on their required classes. We were surprised more didn’t quit when they weren’t chosen for the museum internship and we didn’t notice any divisions in later sections between the students that went to the museum and those that did not. The third cohort lost two additional students at the end of the second semester when one graduated early and another was working off-campus for an internship.

#### Data collection training

It is likely that most students will have little to no experience working with specimens or identifying and measuring phenotypic traits, so they need careful training to collect reliable and accurate data. We began each training day with a short (<20-min) lecture and demonstration explaining the various functions of the structures they were measuring, but the rest of the time was spent working with the specimens in pairs learning to take the measurements discussed in the lecture. During the practical sessions the mentors need to be highly interactive, walking around asking and answering questions and checking that the students are taking the measurements correctly. We ended with several days of mock data collection where the students had to take all the measurements on as many different fishes as possible. Without direct access to specimens, this could be replicated using photographs or scans of museum specimens and training the students to take linear measurements using image analysis software or collect shape data by placing morphometric landmarks on homologous points and semi-landmark curves using various geometric morphometric packages. Every student received this training and their performance during the data collection exercises, along with other things like teamwork, ability to ask questions when uncertain and overall interest in the project, helped us to identify who we invited to join the data collection team at the museum. Our top-five tips for training undergraduates to measure phenotypes are included in Box 1.

**Box 1**

Top-five tips for training undergraduates to measure phenotypes

(i) Choose phenotypic traits that are relatively easy to reliably identify and measure. The more difficult the phenotype the more time needed for training and practice.

(ii) Go slow. Start by demonstrating how to handle specimens and use all the equipment necessary for taking the measurements. Depending on the number and type of measurements you may not want to explain all of them at the start. For example, we began with overall size measurements, then divided the fish up into head, body, tail, and fins each of which we covered on a separate day.

(iii) Start with a short lecture/demonstration (<20 min) covering how to identify and measure the phenotypes, as well as the diversity, function, and the hypothesized ecological role of the structures involved and then get the students to work in pairs to take the measurements on a variety of specimens.

(iv) Make sure the students experience the diversity of phenotypes that they will encounter during their data collection, as homologous structures can look very different across taxa.

(v) Have several practice data collection exercises where the students have to take all of the measurements on as many different specimens as possible within a set time period. Multiple mentors should measure these same specimens to provide a set of values that can be compared with the student measurements. This allows you to identify if there are particular traits that are problematic (and if so, provide additional training) and also, which students are able to quickly and reliably take accurate measurements and thus are ready to begin data collection.

#### Hypothesis development

The hypothesis development section consisted of two phases, the first helped the students to develop the necessary skills to use the scientific literature for generating hypotheses (Phase 1) and the second focused on producing and choosing a hypothesis (Phase 2). Before beginning phase 1 we led a brain-storming session of all the factors the students could possibly think that may influence the body shape of fishes. We did this first to remind the students of their ultimate goal of developing and then testing a hypothesis about fish body shape evolution. A large list was generated on the whiteboard and despite most students not having taken an ichthyology or vertebrate anatomy class the students did surprisingly well at generating a comprehensive list. This was perhaps due to their experiences during the data collection training, as our short lectures at the beginning of each training day included some discussion of the hypothesized ecological role of the structures they were learning to measure. The list of factors was saved to act as a starting point for the hypothesis development in phase 2.

#### Phase 1: developing the necessary skills to engage with scientific literature

We began by discussing the differences between scientific questions, hypotheses, and predictions. We also talked about the ways in which students thought scientists got inspiration for their work: reading papers, observing nature, running experiments, deriving mathematical models, talking to other scientists, etc. We focused on reading papers, as it is an essential skill for scientists to develop but undergraduates in their first few years rarely get an opportunity to fully engage with the scientific literature (Hoskins et al. 2011). Over a series of weeks, we led our undergraduate researchers through a set of mini-lectures, discussions, and exercises to train them to use the scientific literature (Table 1).

**Table 1:** Training students to use the scientific literature

Topic	Active participation
What is a scientific paper and why do we write and read them?	Brainstorm what can be gained from reading a scientific paper.
How to read a scientific paper.	Read each section of a relevant article in class and discuss what important knowledge could be gained from it, driven by specific questions posed to the students. See Online Appendix S3 for an example.
How to find scientific papers using Boolean search terms and how to determine if they are from a trustworthy source.	Series of quick-fire exercises to find the most relevant articles on a variety of topics, e.g., find an empirical paper that demonstrates how diet influences body shape in any animal, repeat but now for fish, etc. Students had to develop their own search terms, choose their bibliographic search engine and to be aware of predatory journals, checking the various whitelists and blacklists of journals, e.g., Cabells https://www2.cabells.com.
How to write a hypothesis based on a scientific paper.	Read a relevant article in class and discuss the hypotheses that could be generated from it. Identify whether hypotheses could be tested with the data currently being collected and if not identify what kind of additional experiments or data would be needed to test it. We repeated this with a different paper for homework. Read three abstracts and use the information within them to develop hypotheses that could be tested with the dataset being generated.

Our research focus is macroevolution and the data are at the species-level, so that was the perspective we presented but we always encouraged students to read a wide-variety of papers from microevolution, ecology, and organismal biology, as inspiration can be derived from many sources.

#### Phase 2: developing hypotheses

**Fig. 1:**
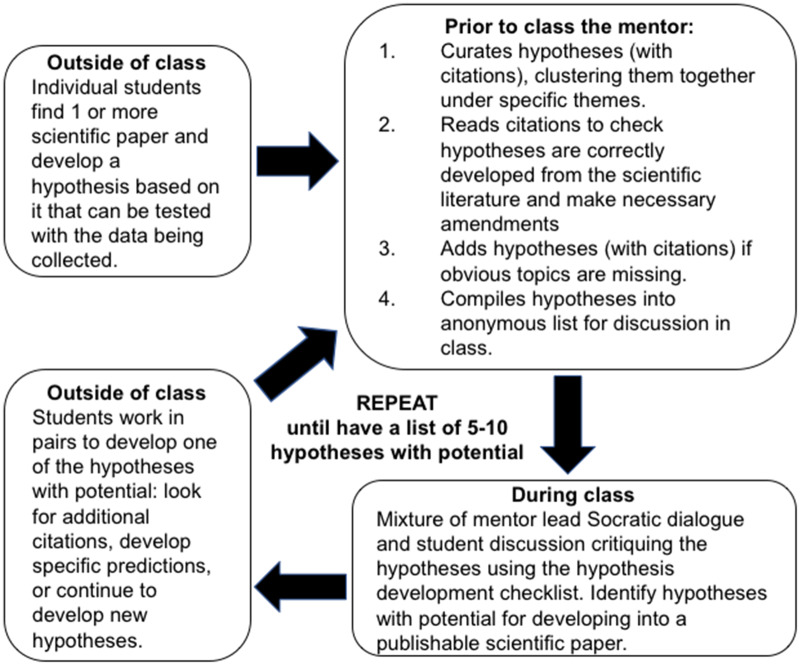
The repeated process of hypothesis development involving students and mentors. It took between 4 and 5 weeks to achieve the goal of 5–10 hypotheses with potential to become the class project.

To help the students to develop and critique the hypotheses, we brainstormed a list of all the things to take into account when developing and choosing a hypothesis or set of hypotheses around which a paper will be written. This hypothesis checklist included critical aspects, such as ensuring the predictions are testable, as well as other factors associated with feasibility and “publishability” (example in Online Appendix S4), which are practical considerations of the process of science that are not talked about explicitly, especially with undergraduates. The students were then tasked with developing a number of hypotheses that they wanted to test using our dataset, along with citations for the articles that they had used to generate each hypothesis (Fig. 1). We suggested they should start by looking for papers that provided support for the factors identified during the initial brainstorming session as possible drivers of body shape change. The process outlined in Fig. 1 was repeated until there were 5–10 hypotheses that had the potential to be developed into a publishable scientific paper. As a group the students then used the hypothesis checklist (Online Appendix S4) to critique hypotheses and then choose two (or more depending on the size of the cohort) to develop into a project proposal. One of the most common critiques concerned the accessibility of additional ecological or environmental data needed to test the hypothesis, particularly as they were under a major time constraint. Once the group had agreed on the two strongest ideas, students assorted themselves into roughly equal sized groups to work on the project proposal presentation. The presentation had to provide appropriate citations and include four sections (i) introduction and background, (ii) hypotheses and predictions, (iii) methods: data and taxonomic scope, and (iv) conclusions: assessment of feasibility and “publishability.” It was particularly important that the students identified the taxonomic scope of the project, as the comparative method relies on having multiple independent evolutionary events and may determine feasibility if they needed to collect additional ecological/environmental data to test their hypothesis. Students were given no restrictions on the use of the dataset; they could develop hypotheses within specific clades or across all of teleosts, although the amount of data available to each cohort varied. The first cohort only had access to the clades we measured during the first summer (Ovalentaria, Carangimorpharia, and Anabantaria), the second cohort had access these as well as most of Eupercaria, and the third had access to all 6000+ species. At the end of the presentations, each student filled out written evaluations of the two projects, and through discussion the group generated a table of the strengths and weaknesses of each proposal. The mentors supplied additional points made in the written assessments (if the student was quiet) or asked questions about particular strengths and weaknesses, if they thought the class was missing something important. Once the group felt they were ready, a vote was taken, and a project chosen.

During the hypothesis development process, we found that the single most important thing we did was generate the anonymous list of hypotheses (Fig. 1). This is because it allowed: (i) students to not feel awkward about directly critiquing another student’s idea, (ii) students that didn’t like to talk in class to still participate in suggesting hypotheses, and (iii) mentors to read the hypotheses and if there were obvious gaps the students weren’t exploring, to add a few hypotheses to the list that could be critiqued by the class without the bias of knowing it was included by their mentors.

This was by far the most difficult aspect of the experience, oftentimes students struggled to identify appropriate hypotheses that could be tested with the morphological data they had collected as part our larger scientific enterprise to understand the drivers of teleostean body shape evolution. It also seemed that students felt out of their depth, not sure if they were doing it right. Some asked for feedback on their hypotheses before the submission deadline and others just appeared to be slightly frustrated by the experience. We quickly noticed students had a tendency to ignore simple hypotheses, such as “habitat influences body shape,” most likely because they thought these were not clever enough and favored convoluted and complex ideas that were difficult to test, especially in the given timeframe. However, as the process progressed, and they were able to begin to discuss and critique the ideas with the checklist, they were able to identify which hypotheses were testable and feasible. Therefore, even if the hypothesis they chose was one the mentors initially added to the anonymous list the students recognized its potential and developed it further.

#### Analytical methods training

Quantitative ability is critical for biological discovery and widely recognized as important for success in medical (Alpern et al. 2009) and graduate biology programs but undergraduates in biological sciences often don’t receive appropriate training (Bialek and Botstein 2004; Barraquand et al. 2014). We chose to use the R software environment for statistical computing (R Core Team 2019) to implement our analytical methods training for a number of reasons. First, it provides the opportunity to train the students in basic command-line and computer programming concepts, which are an essential part of modern biology that needs to be more widely incorporated into the undergraduate biology curriculum (e.g., Pevzner and Shamir 2009; Tan et al. 2009; Goth 2010; Libeskind-Hadas and Bush 2013). Additionally, R is freely available, so the students will be able to continue using it after they graduate from the university moreover the development of coding skills is transferable to many fields of research and employment. R is also commonly used by ecologists (Lai et al. 2019) and evolutionary biologists. In particular, the specific the macroevolutionary hypotheses our students were developing involved modern phylogenetic comparative methods, many of which are available in R (Paradis 2011, https://CRAN.R-project.org/view=Phylogenetics).

Time constraints precluded teaching a full statistics course, so we focused on visualization, analytical reasoning, data interpretation, and understanding basic programming concepts. The majority of students had no experience with any programming language or command line programs, so we went very slowly. The first day was an introduction to the R interface, basic mathematical functions, how data are stored in objects, and the types of object. This was followed by a day on introductory plotting and another on basic statistics. We then had a free day for the students to apply all they had learned so far to exploring their hypothesis. We then moved on to visualizing data on phylogenies, and several weeks of phylogenetic comparative methods, focusing on the reasons why we need to use a phylogeny for statistical analyses across species. We always ended with the undergraduate researchers running the specific phylogenetic comparative analysis needed for their studies, with a basic understanding of how to read and interpret the output. Toward the end of the analytical methods training, it was also helpful to work with the students to generate a workflow for all the steps in the analysis that they had learned, either as R code or a summary diagram. Our annotated R code from the last iteration of the experience is provided as example in Online Appendix S5, subdivided into 90-min sessions.

In our experience this section was the most variable in terms of student engagement. The realization of the importance and power of knowing how to use R occurred at different times. For some, it was when they added data to a phylogeny and made figures that looked like ones in the papers they had read and could start making evolutionary inferences. For others it wasn’t until they actually analyzed the full dataset and generated their own results for their presentations that they realized how much they had learned. At the end of the experience not every student felt comfortable in R. For some, the command-line remained an obstacle and they didn’t understand how objects were stored and could be accessed and manipulated, so they struggled throughout. However, the majority of the class were able to use R to make their own graphs and run analyses for their presentations. Our top-10 tips for teaching analytical methods in “R” are given in Box 2.

**Box 2**

Top-10 tips for teaching R to undergraduates

(i) At the very start spend time explaining about files and folder systems. We quickly realized that students aren’t familiar with setting up a folder and storing all of their documents (datasets and code) in one place, so they can find what they did in previous classes. Also, make sure they are not just typing into the R console but are saving an executable R script.

(ii) Provide an R script document that contains all the notes for that day but with gaps to fill in with code as you go along. Share the full script with the code afterward so they have one that runs with no mistakes, which they can use for future reference and to understand where they made mistakes in their own code.

(iii) Spend time at the beginning explaining how the data are represented in vectors, dataframes, matrices, and lists, making sure to show them how to access data from each. If they can’t do this, then they will not be able to independently write code to run analyses. Keep reiterating this point throughout.

(iv) Go over all jargon/terms in detail and repeat them. Many of the terms (e.g., function, package, object, class, library, etc.) are completely new to students and can be a barrier to learning and comprehension.

(v) Go slow, no matter how much you think you will cover, you probably won’t be able to do it all!

(vi) Use real data so the students are making discoveries as they go along and can start to interpret the results too. It also reminds them why they are learning to use R and keeps them focused on their questions.

(vii) Start each day with follow-along code demonstrations, interspersed with mini-lectures if there is a topic that needs explanation. Explain absolutely everything you are doing and deliberately make mistakes so that students can learn how to debug issues and not just give up because “I’m useless with computers”. Afterward get students to suggest how they might repeat what they have just done on a different column of data or what the problem might be with the code. Next, give them an exercise using exactly the same techniques you demonstrated but with a new dataset or different columns in the dataset.

(viii) Have at least two mentors experienced with R regardless of how small your class is. One instructor should be leading the class through the exercises and the other acts as the “TA” on the look-out for anyone struggling during the follow-along sections, as it is so easy to make a typo and not see where the problem is and get behind. It is also helpful to make the sessions as interactive as possible, and to ask questions about the results that the students are generating on their personal computers. This helps to ensure that students are not left behind because some are reticent to let the instructors know when they encounter errors in their code. Both mentors should be walking around and talking to the groups during exercises in order to identify anyone having problems and help trouble-shoot issues.

(ix) Allow students to work in pairs; many that felt uneasy with programming and took comfort in working with someone else, realizing others were struggling too.

(x) Focus on visualization, as a lot can be inferred from interpreting basic graphs and data plots. In macroevolutionary analyses, morphospaces (scatter plots with each axis representing a morphological variable, like an individual trait, ratio, or principal component analysis axis, which describes some character of the organism) with phylogenetic relationships linking the species (phylomorphospaces) and phylogenies with trait data plotted at the tips, are particularly useful for making evolutionary inferences.

#### Interpreting and presenting results

Depending on the research question, sometimes the students were able to run all of the initial visualizations and preliminary analyses themselves and other times the analyses to test their hypothesis were too computationally intensive, so we had to run them on the high-performance computing (HPC) cluster. In the latter case we would set-up, run, and compile the output from the complex HPC batches and then return the data to the students. The students would interpret the raw output from the analyses or if the complexity of the output was too great, we would generate more readable output such as simplified tables or figures that we would present to the class. The students would discuss their interpretation of each result, whether they supported or contradicted their predictions.

The students were strongly encouraged to present their preliminary findings as either a poster or a talk at a local student conference. All students that participated in the experience over the 3 years chose to present their research and most picked to work in pairs. As a group, we figured out how to split up the research topic, so that each student would be presenting on something slightly different, but we did always talk to the conference organizers to make sure we could give consecutive talks. In total, 14 talks and 4 posters were given over the 3 years by undergraduate researchers, either at the UC Davis Undergraduate Research, Scholarship and Creative Activities Conference or the Clemson Biological Sciences Annual Student Symposium. While preparing for the presentations we emphasized the power of storytelling for communicating science (e.g., Dahlstrom 2014; Olson 2015). Because we had no control over when the conferences were held, it was often a bit of a rush to generate preliminary results to present but it was worth it, as it was the point at which the students took full ownership of the research and realized how much they had learned. Preparing for the talk or poster made the students synthesize all that they had learned throughout the experience and revisit the hypotheses and background literature to construct a narrative. Moreover, for many students the presentation was a highlight of the research experience, as it gave them the opportunity to demonstrate their mastery of the subject to the community. We think it is particularly important that students are given the opportunity to share what they have been working on for the past year or more with friends and family, who may not have any experience with scientific research. Based on our observations, we believe that this chance to celebrate their success with their friends and family may be especially impactful for first generation students.

Following their presentations, the students discussed their preliminary results: how robust they thought they were, whether they were concerned about any issues with the data or analyses, and whether we need to add more analyses or check the data. When necessary, the additional analyses were run and discussed. Not every project led to publishable results but for those that did, the scientific paper was written after the CURE was completed, with one of the mentors leading the writing but with input from the students. In one case we are still waiting on the final analyses, as we needed the completed dataset and the necessary computational resources have been difficult to acquire.

For every cohort, we also included at least one professional development discussion. Depending on interests, we provided a panel of graduate students, PIs, postdoctoral researchers, and lab managers to talk about academic careers and graduate school or led a brain-storming session on how to write about the experience in their applications to jobs or school. The latter was particularly useful for many of the students that were interested in pursuing careers in medical fields, as the research topic did not have a medical focus. It helped to identify all of the transferable skills they developed through the experience.

### Assessing student experience

To assess the impact that the experience had on students we used a survey loosely based on the classroom undergraduate research experience survey (Lopatto and Tobias 2010) that asked students to assess their research skills, opinions on science, and future plans. We asked a series of multiple-choice questions, phrased as statements that they could “strongly disagree,” “disagree,” “not sure/neutral,” “agree,” or “strongly agree” with, as well as free response questions. However, these types of survey have limitations, and responses should be interpreted with caution (Auchincloss et al. 2014). For example, self-reported perceptions of learning show that students in active classrooms feel like they learn less when they are actually learning more (Deslauriers et al. 2019). The first year we attempted to quantify the change in student’s self-assessed skills with a pre- and post-course survey but the student responses to the pre-survey concerning their current skills left little to no room to show any improvement. We therefore relied on a post-course survey that asked the students to assess their current skills and knowledge, as well as the impact the research experience has had on them (see Online Appendix S6 for survey). The survey also contained a section comprised of open questions about their research experience.

The research experience had a positive impact on the student’s self-reported research skills, understanding of the scientific process and future plans (see the table in Online Appendix S7). In terms of skills acquired, one of the best supported and most gratifying trends from the surveys was that the students felt they had greatly improved in their critical thinking abilities and their understanding of the scientific process throughout this experience. In the free responses and discussions with students many of the them also expressed their appreciation for learning basic programming skills. It was somewhat surprising to find that 18% of students disagreed to some extent or were unsure about gaining satisfaction from independent problem solving in science. In terms of the impact that the experience had on student’s career plans, we were pleasantly surprised to find that 59% of students agreed or strongly agreed that the experience “Increased the likelihood that [they] will apply to graduate school specifically for a Ph.D. or Masters in a scientific discipline” and 65% agreed or strongly agreed it “Increased the likelihood that [they] will pursue a career that involves scientific research”.

In the written responses to questions such as “What would you recommend to improve the experience?” and “Are there topics etc. that you wish we had covered or covered in more detail?” The most common answers were that they would have enjoyed additional training in statistical analyses in R, as it felt rushed at times and also that it would be profitable to spend more time working on scientific writing skills.

## Future developments

Now that we have finalized the traditional (linear) morphometrics database from the museum, the data-collection is switching to analyzing the photographs using geometric morphometrics. We are also experimenting with streamlining the experience so that students only have to commit to two semesters (12 months), as many undergraduates are unable to commit to three semesters or four quarters working on a research project. For example, we are now providing a specific clade and broad topic to work on, which should speed up the hypothesis development, data collection, and analysis sections. This approach will also focus students’ literature searches and shorten the time spent becoming familiar with the literature and developing their own potentially publishable hypothesis. As the students will not be working on the full dataset going forward, it will also speed up the collection of any additional data they need (e.g., diet or habitat information) and reduce computational demands, as they will only have 200–300 species to analyze, versus thousands.

We have also recently realized how important it is to openly acknowledge at the start of the process that it will be hard, and that sometimes they may feel clueless but that those feelings are both normal and okay. For example, students have to choose a hypothesis before they know any comparative methods, so they have to trust us that there are methods available to test the hypothesis until we can teach them the required quantitative skills. We now emphasize that as they progress through the experience things will become clearer and they should ask as many questions as possible to get clarification, help, and advice; perseverance is key. This is why we believe preparing for the presentation of their results is a pivotal point in the experience: by synthesizing everything they have done the students finally take ownership of the research and realize just how far they have come.

## Conclusions

Careful training of undergraduate researchers to collect phenotypic data in the context of a CURE has major scientific and pedagogical benefits. Our work demonstrates that this strategy enables the collection of vast amounts of phenotypic data by generating a workforce of knowledgeable and committed undergraduate scientists with a vested interest in the data quality. Moreover, it provides the undergraduate researchers with the opportunity to experience the entire process of science, from hypothesis development through to the presentation of results. Through this experience the students build key skills such as critical thinking, quantitative reasoning, and public speaking, all invaluable tools for modern scientific discovery.

## Supplementary Material

obaa004_Supplementary_Data
