# Supplementary material for: A CURE for a Major Challenge in Phenomics: A Practical Guide to Implementing a Quantitative Specimen-Based Undergraduate Research Experience
Source: Integr Org Biol. 2020 Feb 20;2(1):obaa004. doi: 10.1093/iob/obaa004 (PMC7671122; doi:10.1093/iob/obaa004)
Supplement: obaa004_Supplementary_Data [file obaa004_supplementary_data.zip › Appendix1.pdf]

Appendix 1: potential resources for specimen images, phenotypic data and morphometric software.

Note, when using images from multiple sources you need to ensure that the images are consistent, showing the same view and taken at the same angle and ideally containing a scale bar or taken at the same distance from the specimen.

Specimen images

1) Photographs
   - MorphBank http://www.morphbank.net/About/AboutMb/
   - MorphoBank https://morphobank.org/
   - iDigBio https://www.idigbio.org/
   - PlantImageAnalysis https://www.plant-image-analysis.org/dataset
   - Smithsonian National Museum of Natural History online collections https://collections.nmnh.si.edu/search/
   - Natural History museum London UK data collection https://data.nhm.ac.uk/
   - Teleost fish opercle bone images https://datadryad.org/stash/dataset/doi:10.5061/dryad.d3h31
   - John E. Randall's reef fish photographs http://pbs.bishopmuseum.org/images/JER/
   - New York Botanical garden C.V. Starr virtual herbarium http://sweetgum.nybg.org/science/vh/
   - SouthEast Regional Network of Expertise and Collections herbaria http://sernecportal.org/portal/

2) 3D scans
   - MorphoSource https://www.morphosource.org/About/home
   - Phenome10K https://phenome10k.org/
   - DigiMorph http://digimorph.org/
   - Digital Life 3D https://sketchfab.com/DigitalLife3D/models

Phenotypic data

1. TRY: plant trait database https://www.try-db.org/TryWeb/Home.php
2. EltonTraits 1.0: Species-level foraging attributes of the world's birds and mammals http://www.esapubs.org/archive/ecol/E095/178/

3. PanTHERIA: a species-level database of life history, ecology, and geography of extant and recently extinct mammals http://esapubs.org/archive/ecol/E090/184/
4. Amniote life history database http://www.esapubs.org/archive/ecol/E096/269/
5. AnAge: a curated database of ageing and life history in animals, http://genomics.senescence.info/species/
6. Angiosperm vessel anatomy https://datadryad.org/stash/dataset/doi:10.5061/dryad.1138
7. William W. Howells human craniometric dataset http://web.utk.edu/~auerbach/HOWL.htm
8. Size data on elaphid snakes https://datadryad.org/stash/dataset/doi:10.5061/dryad.cr788
9. Geometric morphometric landmark data on lateral photographs of John E. Randall's reef fish photographs https://datadryad.org/stash/dataset/doi:10.5061/dryad.gh4k7
10. User averaged landmark configurations of 3D scanned bird beaks https://datadryad.org/stash/dataset/doi:10.5061/dryad.4006fm8
11. Vertebrate body and brain mass https://figshare.com/articles/Brain_mass_and_body_mass_datasets_and_phylogenies_linked_to_brain-body_allometry_and_the_encephalization_of_birds_and_mammals/6803276/1
12. Floral reflectance database http://www.reflectance.co.uk/

There are many more inter-specific and intra-specific datasets listed on this blog page: https://ramblingsofanecologa.wordpress.com/2016/07/26/are-these-the-data-you-are-looking-for/ .The British Ecological Society Macroecology group are also trying to curate a list here: https://docs.google.com/spreadsheets/d/1lZ8XzhEnXOlkvepkxOnGvtRr3gTSNm3hlkRgELxfTLI/edit#gid=1150363964

Thanks to everyone on twitter for responding to my appeal for data sources!

Morphometric software

1) Traditional linear and area measurements
    - ImageJ https://imagej.nih.gov/ij/index.html a simple to use standalone Graphical User Interface that will allow you to take linear and area measurements etc. on 2D images. Tutorials can be accessed here: https://imagej.nih.gov/ij/docs/examples/index.html

2) Geometric Morphometrics
    - stereomorph https://cran.r-project.org/web/packages/StereoMorph/index.html an R package can place landmarks and semi-landmarks on 2D and stereo camera images using the *digitizeImages* function. A user guide can be found here: https://aaronolsen.github.io/tutorials/stereomorph.html

- geomorph https://cran.r-project.org/web/packages/geomorph/index.html an R package for collection and analysis of geometric morphometric data. Can place landmarks on 2D images using the *digitize2d* function and semi-landmarks using the *define.sliders.2d* function. Can place landmarks on 3D images using the *digit.fixed* function or an interactive template of three-dimensional surface sliding semi-landmarks can be built using the *buildtemplate* function. A user guide can be found here: http://www.emmasherratt.com/uploads/2/1/6/0/21606686/quick_guide_to_geomorph-_introduction.html
- tpsDIG2 https://life.bio.sunysb.edu/morph/soft-dataacq.html a Windows only standalone program for the placement of landmarks and semi-landmarks.
- 3D-Slicer https://download.slicer.org/ and extensions https://slicermorph.github.io/ used for placing landmarks on CT-scans and surface 3D scans.
