# Supplementary material for: A CURE for a Major Challenge in Phenomics: A Practical Guide to Implementing a Quantitative Specimen-Based Undergraduate Research Experience
Source: Integr Org Biol. 2020 Feb 20;2(1):obaa004. doi: 10.1093/iob/obaa004 (PMC7671122; doi:10.1093/iob/obaa004)

Appendix 2: CURE timeline for the UC Davis quarter system and Clemson semester system.

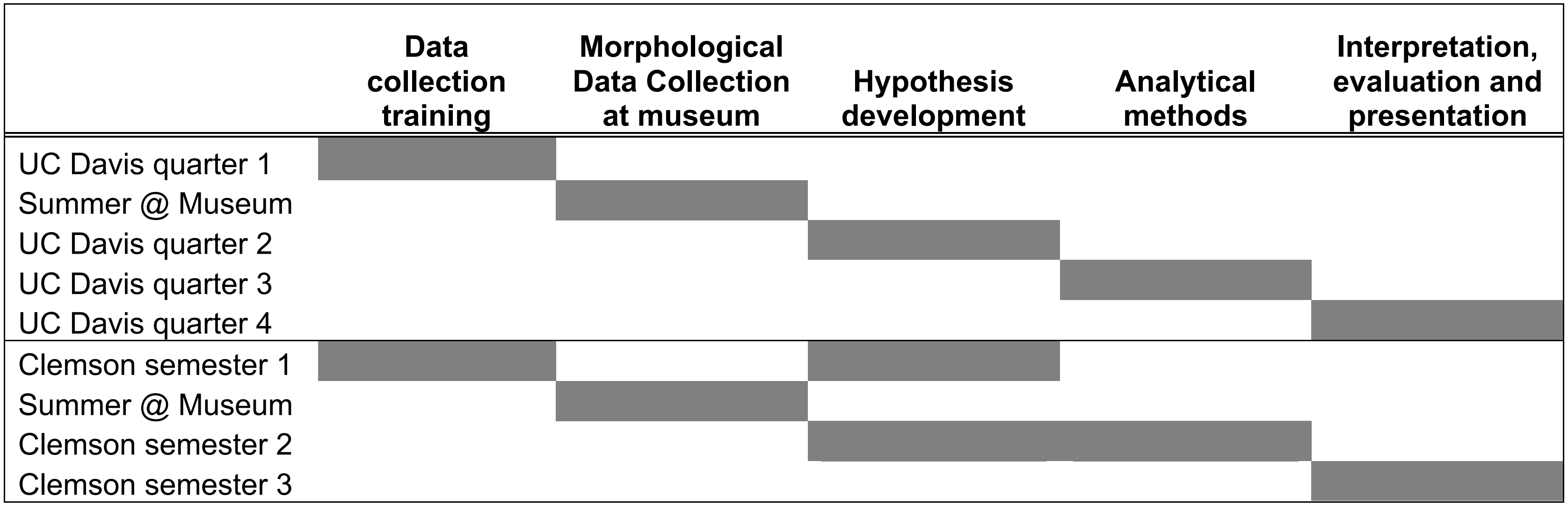

Supplement: obaa004_Supplementary_Data [file obaa004_supplementary_data.zip › Appendix2.pdf]
