# Supplementary material for: A CURE for a Major Challenge in Phenomics: A Practical Guide to Implementing a Quantitative Specimen-Based Undergraduate Research Experience
Source: Integr Org Biol. 2020 Feb 20;2(1):obaa004. doi: 10.1093/iob/obaa004 (PMC7671122; doi:10.1093/iob/obaa004)
Supplement: obaa004_Supplementary_Data [file obaa004_supplementary_data.zip › Appendix3.pdf]

Appendix 2 – example of critically reading scientific papers

We read *Tsuboi, M., Gonzalez-Voyer, A., & Kolm, N. (2015). Functional coupling constrains craniofacial diversification in Lake Tanganyika cichlids. Biology letters, 11(5), 20141053* in class going through all these questions. This paper was chosen as it is highly relevant to our work, i.e. it illustrates one of the types of macroevolutionary questions we can easily ask with our data and it is short simple paper focusing on a single question. We started with the Introduction and explained how to read the paper critically and creatively, as not everything in a scientific article is stated explicitly. After we had read the introduction, we asked them three questions relevant to the Introduction:

INTRODUCTION

1) What is the 'big picture' question this research addresses? Why is the research important? (e.g. What is its purpose? Is there a specific problem or knowledge gap this research addresses?)
2) What is the background context for this study? Additionally, identify two important references that you could read for further context on this topic.
3) What are the specific questions and hypotheses addressed in this study?

After that we deconstructed the second paragraph, by breaking it down into sentences and asking the students what comments and questions each brought to mind (examples in blue italics*)*. We explained you can improve your understanding by actively drawing inferences from the material using your prior knowledge or by asking questions and looking up the answers to them in other sources. The first paragraph of the introduction was not appropriate for this exercise, as it is an explanation of the concept of functional decoupling.

<u>Second paragraph of Tsuboi et al. 2015 with examples of the types of questions we can ask ourselves after each sentence.</u>

Mouthbrooding in teleost fishes has evolved from an ancestral state of substrate guarding, in which parents typically spawn and guard their eggs on a sand substrate or stone substrate or in rock holes/crevices *Comment: If mouthbrooding is a derived state, what about other forms of parental care like nest building? How widespread is the evolution of substrate guarding in fishes? Has parental care evolved many times? What kinds of parental care are there across Teleost fishes?* The evolutionary transition to mouthbrooding offers an excellent opportunity to test the functional coupling hypothesis because mouthbrooding introduces a novel function to the cranium, which is originally adapted for feeding *Comment: What is the evidence that mouthbrooding alters the selective pressures on the cranium? What other parts of the anatomy may be under selective pressures due to mouthbrooding? Also, some fish can use their mouths for ventilation…is this system also affected by mouthbrooding?* Previous studies have found that uni-parental mouthbrooding is often accompanied by sexual dimorphism in craniofacial anatomy. Additionally, a trade-off between reproduction and feeding was reported in cardinalfishes and cichlids, suggesting that the functional coupling of feeding and brooding may impede morphological diversification. *Comment: Okay this answers*

my first question about the evidence that mouthbrooding alters the selective pressures on the cranium. So, since mouthbrooding impedes diversification, I wonder why it has evolved multiple times. Is there any convergence in ecology (habitat, diet, etc.) i.e. a pre-adaptation, which allows the evolution of mouthbrooding.

We then went on to read each additional section of the paper and asking the students the questions below:

METHODS

4) What is the sample size and what types of data are collected (e.g. body length or tooth shape)? What is the breadth of the study in terms of taxonomic rank (e.g. within a single Order, Family, Genus or species or community)?

RESULTS

5) What are the major findings of the study? Do these results support or contradict the authors' expectations?

DISCUSSION

6) What are the authors' explanations or interpretations of their findings?
7) Following on from this study what additional questions should be investigated?

OTHER

8) How can the knowledge you have gained from this paper be applied to our data on fish body shapes? Are there specific questions/hypotheses that come to mind?
9) What are the key references in this paper?
10) List up to three words or concepts you had to lookup definitions for.
