# Supplementary material for: A CURE for a Major Challenge in Phenomics: A Practical Guide to Implementing a Quantitative Specimen-Based Undergraduate Research Experience
Source: Integr Org Biol. 2020 Feb 20;2(1):obaa004. doi: 10.1093/iob/obaa004 (PMC7671122; doi:10.1093/iob/obaa004)
Supplement: obaa004_Supplementary_Data [file obaa004_supplementary_data.zip › Appendix4.docx]

**Appendix 4: Hypothesis Check list**

Criteria for evaluating scientific hypotheses

This list is a set of guidelines for identifying interesting and publishable scientific questions. It takes the form of a set of questions, ranging from critical to trivial, you should ask yourself when formulating your hypotheses. *Points in italics are specific to our project asking comparative evolutionary questions.*

**Critical**

- Are your hypotheses and predictions testable? i.e. are counter examples logically possible?
- Is there citable background information/context (previous studies or theory) to justify your question and explain your predictions?
- Replication – how many replicates do you need? In experimental systems you run the experiment several times – the exact number of replicates necessary to identify statistically significant trends depends on the study design. *In our comparative phylogenetic framework we ideally need evolutionary replicates, which means checking that our character of interest e.g. mouth brooding, has evolved more than once in our group of interest. However, as we have seen if the question and result is of sufficient interest it is not strictly necessary (e.g. mouth brooding in cichlids Tsuboi et al. 2015).*

**Feasibility**

- Do you have the ability to collect the necessary data? Think about:
  - Measurability – is the character well defined? e.g. Intelligence vs body weight.
  - Availability – are data readily available? For example, you would not want do a broad study comparing the diets of deep sea fishes since we don’t have dietary data on most deep sea species.
  - Accessibility – do you have access/rights to these data?
  - Comparability – if you are combining data from different sources, are they equivalent? For example, maximum lengths for species in FishBase may refer to standard length or total length.
- Do you have adequate time and resources to study this question?
  - Be cognizant of scope and taxonomic scale
  - Be aware that many broad scale questions require a lot of computational power to analyze the data.
- Do the methods currently exist to answer your question?
- Do you have an adequate sample size to identify statistically significant results?
- Are the data you will be using reliable? For example, species identifications on GenBank gene sequences may be incorrect at times.

**‘Publishability’**

Knowing whether a project stands a good chance of being published in a scientific journal takes experience and relies a lot on knowledge of intangible issues concerning the degree of interest in the topic. In reality it also involves a degree of luck, as just 2-3 reviewers get to decide the fate of your paper at a journal although picking the right journal with editors that understand your research can ameliorate this issue. There are quite a few things you should think about that all contribute to the likelihood of getting your study published.

- Is it concise? Limit your question to a few, clear predictions as long rambling papers are hard to understand and hard to get published.
- Applications – to current issues in fisheries or climate change
- Predictive ability – do you generate lots of opportunities for new studies?
- Has this exact study been conducted before? Investigating previously investigated questions in novel groups, systems or using new methods, etc. is acceptable but a duplicated study will not get published.
- Publishing norms emphasize novel, positive results. Does your study generate novel and interesting results regardless of whether the hypothesis is supported or rejected? This is the holy grail of hypotheses!
- Is your question sufficiently interesting?
  - - Who is your target audience? The scope of your topic will dictate interest level and determine your target journals for publication. Specific (specialized, smaller, lower impact journals) 🡪 General (integrative, bigger, higher impact journals).
    - More novel questions may fill an important knowledge gap and hence be of wide interest. However, if you fail to justify your novel question based on existing work people will struggle to put it into context and will likely reject it.
    - Are your species charismatic? For example, the general public would be more interested in a study on sharks than the same study done on goldfish. However, people who study cyprinids would likely read your goldfish study.
    - Controversial questions can be good or bad depending on the question and your findings.
