# Supplementary material for: A CURE for a Major Challenge in Phenomics: A Practical Guide to Implementing a Quantitative Specimen-Based Undergraduate Research Experience
Source: Integr Org Biol. 2020 Feb 20;2(1):obaa004. doi: 10.1093/iob/obaa004 (PMC7671122; doi:10.1093/iob/obaa004)
Supplement: obaa004_Supplementary_Data [file obaa004_supplementary_data.zip › Appendix6.docx]

**Appendix 6: Final Survey – Research Experience**

**SECTION A – YOUR HONEST ASSESSMENT OF YOUR CURRENT skills, understanding of science and future plans**

**1. Please rate your level of agreement with the following statements regarding your current research skills**

|  | Strongly disagree | Disagree | Not sure | Agree | Strongly agree |
| --- | --- | --- | --- | --- | --- |
| I can work effectively in multidisciplinary and diverse groups |  |  |  |  |  |
| I can provide unbiased and constructive criticism to other students |  |  |  |  |  |
| I can search for and locate primary scientific literature relevant to a specific topic |  |  |  |  |  |
| I can read and understand primary scientific literature (scientific research articles) |  |  |  |  |  |
| I can develop a robust and testable scientific hypothesis |  |  |  |  |  |
| I can effectively apply the scientific process and develop a procedure to address a research problem |  |  |  |  |  |
| I can implement statistical analyses of data |  |  |  |  |  |
| I understand basic computer programming concepts |  |  |  |  |  |
| I am good at interpreting data generated from analytical procedures |  |  |  |  |  |
| I know how to use figures, graphs, charts, tables and drawings to effectively communicate research findings |  |  |  |  |  |
| I can present results in written papers or reports |  |  |  |  |  |
| I can present results orally |  |  |  |  |  |
| I can interpret data while relating results to the original research question/hypothesis |  |  |  |  |  |
| I can express why my results are an important contribution to my field |  |  |  |  |  |
| I can summarize complex results by emphasizing what is most important to the story |  |  |  |  |  |
| I appreciate the value of constructive criticism from my teammates and peers |  |  |  |  |  |
|  | Strongly disagree | Disagree | Not sure | Agree | Strongly agree |
| I can express why my results are an important contribution to my field |  |  |  |  |  |
| I can develop a research question based on current research in my field |  |  |  |  |  |
|  |  |  |  |  |  |

**2. Please rate your level of agreement with the following statements regarding yourself and science**

|  | Strongly disagree | Disagree | Not sure | Agree | Strongly agree |
| --- | --- | --- | --- | --- | --- |
| Creativity does not play a role in science |  |  |  |  |  |
| Science is essentially an accumulation of facts, rules, and formulas. |  |  |  |  |  |
| If the null hypothesis can’t be rejected, the research was a failure. |  |  |  |  |  |
| I get personal satisfaction when I solve a scientific problem by figuring it out myself. |  |  |  |  |  |
| I am self-confident about my ability to do research |  |  |  |  |  |
| I am persistent when I encounter obstacles in my scientific endeavours |  |  |  |  |  |

**3. Please rate your level of agreement with the following statements regarding your future plans**

|  | Strongly disagree | Disagree | Not sure | Agree | Strongly agree |
| --- | --- | --- | --- | --- | --- |
| I am considering a career that would involve research |  |  |  |  |  |
| I am considering attending graduate school |  |  |  |  |  |
| I am considering attending graduate school where I would be doing research |  |  |  |  |  |

**SECTION B – YOUR HONEST ASSESSMENT OF THE IMPACT THAT THIS RESEARCH EXPERIENCE HAS ON YOUR skills, understanding of science and future plans**

**4. Please rate your level of agreement with the following statements regarding the impact this research experience has had on your skills**

|  | Strongly disagree | Disagree | Not sure | Agree | Strongly agree |
| --- | --- | --- | --- | --- | --- |
| Improved my ability to work effectively in multidisciplinary and diverse groups |  |  |  |  |  |
| Improved my ability to provide unbiased and constructive criticism to other students |  |  |  |  |  |
| Improved my ability to find relevant scientific papers |  |  |  |  |  |
| Improved my ability read and understand primary scientific literature (scientific research articles) |  |  |  |  |  |
| Improved my ability to develop a robust and testable scientific hypotheses |  |  |  |  |  |
| Improved my ability to effectively apply the scientific process and develop a procedure to address a research problem |  |  |  |  |  |
| Improved my ability to implement statistical analyses of data |  |  |  |  |  |
| Improved my ability to understand basic computer programming concepts |  |  |  |  |  |
| Improved my ability to interpret data generated from analytical procedures |  |  |  |  |  |
| Improved my ability to use figures, graphs, charts, tables and drawings to effectively communicate research findings |  |  |  |  |  |
| Improved my ability to present results in written papers or reports |  |  |  |  |  |
| Improved my ability to present results orally |  |  |  |  |  |
| Improved my ability to think critically about science and the scientific literature. |  |  |  |  |  |
| Throughout my participation in this activity, I developed a feeling of ownership of the project |  |  |  |  |  |

**5. Please rate your level of agreement with the following statements regarding the impact this research experience has had on your understanding of science and the scientific process**

|  | Strongly disagree | Disagree | Not sure | Agree | Strongly agree |
| --- | --- | --- | --- | --- | --- |
| I have a better understanding of the process of science |  |  |  |  |  |
| I am better equipped think about and critically assess scientific research |  |  |  |  |  |
| I am more disillusioned and confused about science and the scientific process |  |  |  |  |  |
| I have a better understanding of what pursuing a graduate degree in a scientific discipline involves |  |  |  |  |  |

**6. Please rate your level of agreement with the following statements regarding the impact this research experience has had on your future plans**

|  | Strongly disagree | Disagree | Not sure | Agree | Strongly agree |
| --- | --- | --- | --- | --- | --- |
| Increased the likelihood that I will pursue further research opportunities during my undergraduate degree (leave blank if you are graduating this quarter!) |  |  |  |  |  |
| Helped me to narrow down my future career goals |  |  |  |  |  |
| Increased the likelihood that I will pursue a career that involves scientific research |  |  |  |  |  |
| Increased the likelihood that I will apply to graduate school |  |  |  |  |  |
| Increased the likelihood that I will apply to graduate school specifically for a Ph.D or Masters in a scientific discipline |  |  |  |  |  |
| Helped me to realise that I don’t want to pursue a scientific career |  |  |  |  |  |

**SECTION C – FEEDBACK your opportunity to expand upon your experiences.**

**7. What do you wish you knew about the experience at the beginning?**

**8. What do you value most about this research experience?**

**9. Did you like working as a member of a team? And has the group influence extended beyond the research experience – for example have you worked with anyone or got help/advice from anyone in the group about other classes etc.?**

**10. Did you receive enough help and support throughout the experience to develop, test and present your research?**

**11. What would you recommend to improve the experience?**

**12. Are there topics etc. that you wish we had covered or covered in more detail?**

**13. Any other comments, thoughts or ideas you would like to share with us about this research experience.**
