# Supplementary material for: A CURE for a Major Challenge in Phenomics: A Practical Guide to Implementing a Quantitative Specimen-Based Undergraduate Research Experience
Source: Integr Org Biol. 2020 Feb 20;2(1):obaa004. doi: 10.1093/iob/obaa004 (PMC7671122; doi:10.1093/iob/obaa004)
Supplement: obaa004_Supplementary_Data [file obaa004_supplementary_data.zip › Appendix7.pdf]

Question	Strongly disagree	Disagree	Not sure	Agree	Strongly agree
I can work effectively in multidisciplinary and diverse groups	0	0	0	41.18	58.82
I can provide unbiased and constructive criticism to other students	0	0	0	58.82	41.18
I can search for and locate primary scientific literature relevant to a specific topic	0	0	0	47.06	52.94
I can read and understand primary scientific literature (scientific research articles)	0	0	2.94	64.71	32.35
I can develop a robust and testable scientific hypothesis	0	2.94	8.82	67.65	20.59
I can effectively apply the scientific process and develop a procedure to adress the research problem	0	0	2.94	52.94	44.12
I can implement statistical analyses of data	0	0	6.06	69.7	24.24
I understand basic computer programming concepts	0	3.03	12.12	72.72	12.12
I am good at interpreting data generated from analytical procedures	0	0	12.12	57.58	30.3
I know how to use figures, graphs, charts, tables and drawings to effectively communicate research findings	0	0	0	36.36	63.64
I can present results in written papers and reports	0	3.03	15.15	51.52	30.3
I can present results orally	0	0	6.06	51.52	42.42
I can interpret data while relating results from to the original research question/hypothesis	0	0	0	14.29	85.71
I can express why my results are an important contribution to my field	0	0	0	28.57	71.43
I can summarize complex results by emphasizing what is most important to the story	0	0	0	28.57	71.43
I appreciate the value of constructive criticism from my teammates and peers	0	0	0	14.29	85.71
I can express why my results are an important contribution to my field	0	0	0	28.57	71.43
I can develop a research question based on current research in my field	0	0	14.29	28.57	57.14
Creativity does not play a role in science	69.7	27.27	0	3.03	0
Science is essentially an accumulation of facts, rules and formulas	20.59	50	11.76	17.64	0
If the null hypothesis can't be rejected, the research was a failure	82.35	11.76	2.94	2.94	0
I get personal satisfaction when I solve a scientific problem by figuring it out myself	6.06	9.09	3.03	39.39	42.42
I am self-confident about my ability to do research	0	0	14.29	28.57	57.14
I am persistant when I encounter obstacles in my scientific endeavours	0	0	0	42.86	57.14
I am considering a career that would involve research	0	5.88	26.47	29.41	38.24
I am considering attending graduate school	0	5.88	8.82	32.35	52.94
I am considering attending graduate school where I would be doing research	0	5.88	20.59	32.35	41.18
Improved my ability to work effectively in multidisciplinary and diverse groups	0	0	2.94	47.06	50

Improved my ability to provide unbiased and constructive criticism to other students	0	0	2.94	47.06	50
Improved my ability to find relevant scientific papers	0	0	0	35.29	64.71
Improved my ability to read and understand primary scientific literature (scientific research articles)	0	0	0	35.29	64.71
Improved my ability to develop a robust and testable scientific hypothesis	0	0	2.94	32.35	64.71
Improved my ability to effectively apply the scientific process and develop a procedure to adress a research problem	0	0	2.94	35.29	61.76
Improved my ability to implement statistical analyses of data	0	0	0	38.24	61.76
Improved my ability to understand basic computer programming concepts	0	0	0	35.29	64.71
Improved my ability to interpret data generated from analytical procedures	0	0	0	38.24	61.76
Improved my ability to use figures, graphs, charts, tables and drawings to effectively communicate research findings	0	0	0	26.47	73.53
Improved my ability to present results in written papers and reports	0	5.88	11.76	32.35	50
Improved my ability to present results orally	0	0	0	29.41	70.59
Improved my ability to think critically about science and the scientific literature	0	0	0	0	100
Throughout my participation in this activity, I developed a feeling of ownership of the project	0	0	0	28.57	71.43
I have a better understanding of the process of science	0	0	0	26.47	73.53
I am better equipped to think about and critically assess scientific research	0	0	2.94	29.41	67.65
I am more disillusioned and confused about science and the scientific process	44.12	41.18	5.88	2.94	5.88
I have a better understanding of what pursuing a graduate degree in a scientific discipline involves	0	2.94	5.88	38.24	52.94
Increased likelihood that I will pursue further research opportunities during my undergraduate degree	0	0	0	42.31	57.69
Helped me to narrow down my future carreer goals	0	8.82	26.47	44.12	20.59
Increased the likelihood that I will pursue a career that involves scientific research	0	8.82	23.53	41.18	26.47
Increased the likelihood that I will apply to graduate school	0	0	17.65	50	32.35
Increased the likelihood that I will apply to graduate school specifically for a Ph.D. or Masters in a scientific discipline	2.94	8.82	29.41	26.47	32.35
Helped me to realize tha tI don't want to pursue a scientific career	61.76	29.41	5.88	2.94	0
